# Outcome of Elderly Patients With Surgically Treated Brain Metastases

**DOI:** 10.3389/fonc.2021.713965

**Published:** 2021-07-26

**Authors:** Muriel Heimann, Niklas Schäfer, Christian Bode, Valeri Borger, Lars Eichhorn, Frank A. Giordano, Erdem Güresir, Andreas H. Jacobs, Yon-Dschun Ko, Jennifer Landsberg, Felix Lehmann, Alexander Radbruch, Christina Schaub, Katjana S. Schwab, Johannes Weller, Ulrich Herrlinger, Hartmut Vatter, Patrick Schuss, Matthias Schneider

**Affiliations:** ^1^ Department of Neurosurgery, Center of Integrated Oncology (CIO) Bonn, University Hospital Bonn, Bonn, Germany; ^2^ Division of Clinical Neuro-Oncology, Department of Neurology, Center of Integrated Oncology (CIO) Bonn, University Hospital Bonn, Bonn, Germany; ^3^ Department of Anesthesiology and Intensive Care, University Hospital Bonn, Bonn, Germany; ^4^ Department of Radiation Oncology, Center of Integrated Oncology (CIO) Bonn, University Hospital Bonn, Bonn, Germany; ^5^ Department of Geriatric Medicine and Neurology, Johanniter Hospital Bonn, Bonn, Germany; ^6^ Department of Oncology and Hematology, Center of Integrated Oncology (CIO) Bonn, Johanniter Hospital Bonn, Bonn, Germany; ^7^ Department of Dermatology and Allergy, Center of Integrated Oncology (CIO) Bonn, University Hospital Bonn, Bonn, Germany; ^8^ Department of Neuroradiology, Center of Integrated Oncology (CIO) Bonn, University Hospital Bonn, Bonn, Germany; ^9^ Department of Internal Medicine III, Center of Integrated Oncology (CIO) Bonn, University Hospital Bonn, Bonn, Germany

**Keywords:** geriatric, brain metastasis, frailty, CCI, prognosis, cancer

## Abstract

**Object:**

In the light of an aging population and ongoing advances in cancer control, the optimal management in geriatric patients with brain metastases (BM) poses an increasing challenge, especially due to the scarce data available. We therefore analyzed our institutional data with regard to factors influencing overall survival (OS) in geriatric patients with BM.

**Methods:**

Between 2013 and 2018, patients aged ≥ 65 years with surgically treated BM were included in this retrospective analysis. In search of preoperatively identifiable risk factors for poor OS, in addition to the underlying cancer, the preoperative frailty of patients was analyzed using the modified Frailty Index (mFI).

**Results:**

A total of 180 geriatric patients with surgically treated BM were identified. Geriatric patients categorized as least-frail achieved a median OS of 18 months, whereas frailest patients achieved an OS of only 3 months (p<0.0001). Multivariable cox regression analysis detected “multiple intracranial metastases” (p=0.001), “infratentorial localization” (p=0.011), “preoperative CRP >5 mg/l” (p=0.01) and “frailest patients (mFI ≥ 0.27)” (p=0.002) as predictors for reduced OS in older patients undergoing surgical treatment for BM.

**Conclusions:**

In this retrospective series, pre-operative frailty was associated with poor survival in elderly patients with BM requiring surgery. Our analyses warrant thorough counselling and support of affected elderly patients and their families.

## Introduction

According to standard of care, patients with brain metastases (BM) require multimodal therapy that frequently includes surgical removal of the intracranial mass (1). In addition to radiation therapy, surgical removal of a BM with maximum safety is an important part of treatment standards in BM management (2). Despite established surgical procedures and academic research on risk/benefit considerations, e.g. with regard to postoperative complications, the management of elderly patients with BM remains a challenge due to higher rates of treatment-related toxicities and impaired recovery rates (3, 4), predominantly due to the high prevalence of comorbidities among older adults (5). Another challenge for an optimal treatment of BM in geriatric patients is the balance between achieving surgical radicality and preventing secondary complications, which can have a negative impact on further therapy and thus on OS of these patients (4). For this reason, especially in older patients, certain aspects are important for a better assessment of the (surgical) treatment course/success (6). For example, a more detailed evaluation of physical resources prior to treatment represents one such possibility (7). Frailty has become a generally accepted and widely - used term indicating limited reserves in several organ systems because of accompanying disease, lack of activity inadequate nutrition as well as the physiological changes of aging (8). In order to sufficiently objectify frailty parameters in clinical daily routine, the Canadian Study on Health and Aging has developed a frailty index based on a cumulative deficit model (9). This frailty index was linked to eleven variables from the database of the American Surgeons National Surgical Quality Improvement Program (NSQIP) to create a modified frailty index (mFI) (7, 9). The mFI considers items like diabetes, functional status, history of myocardial infarction, hypertension requiring medication and previous cerebrovascular accident with neurological deficit among others (9) entailing an individual patient-specific value of frailty. However, there is only limited data available covering not only health status and robustness, but also frailty and comorbidity burden and its effects on survival in elderly patients with BM. We therefore analyzed our institutional database with regard to a potential influence of the above-mentioned preoperative parameters on the treatment success in geriatric patients with BM requiring surgery.

## Methods

### Patients

All patients with newly diagnosed BM secondary to different primary cancers operated on at the author’s institution between 2013 and 2018 were reviewed retrospectively. Only patients aged ≥ 65 years who underwent surgery for brain metastases were included in further analysis. Approval for this study was provided by the institutional ethics committee (protocol no. 250/19).

Information such as patient characteristics, radiological attributes, site of primary malignancy, laboratory values, functional status at admission and during follow-up were obtained and compiled into a computerized database (SPSS, version 25, IBM Corp., Armonk, NY). Charlson comorbidity index (CCI) was utilized to estimate the preoperative comorbid disease burden. After age adjustment, elderly patients with BM were categorized into two groups marked by CCI ≥ 10 and CCI < 10, as previously described (4). To classify patients according to their preoperative functional status at admission, Karnofsky Performance Score (KPS) was used. For further analysis regarding the American Society of Anesthesiologists (ASA) classification, the geriatric patients studied were divided into two groups: preoperative ASA 1 or 2 *versus* preoperative ASA ≥ 3. C-reactive protein (CRP) and white blood cell (WBC) laboratory values were obtained within 12 h of admission as part of routine laboratory procedures. WBC counts (normal range 3.9-10.2 G/L) were dichotomized into ≤12 G/L and >12 G/L, and CRP (normal range 0-3 mg/L) was dichotomized into ≤ 5 mg/L and > 5 mg/L. BMs were further categorized based on the primary site of origin: lung, breast, melanoma, gastrointestinal, hematologic, prostate, kidney, or other. All treatment procedures were determined individually for each patient by consensus at a weekly interdisciplinary tumor board meeting, as previously reported (4). Overall survival (OS) was measured from the date of surgery for BM until death or last follow-up. In case of absent outcome, patients were classified as being lost-to-follow-up and excluded from further analysis.

### Modified Frailty Index

The modified Frailty Index (mFI) was used to determine the frailty of the elderly patients studied. The mFI consists of eleven items based on the National Surgical Quality Improvement Program (NSQIP) [see **Supplementary Table S1** (9, 10)]. Each mFI variable is equally weighted and counted as 1. The mFI is then derived for each individual patient from the sum of these separate factors and divided by 11. Based on previous reports, patients were divided into three groups based on their admission mFI: “least-frail” (mFI 0-0.08), “moderately-frail” (0.09-0.26), and “frailest” (mFI ≥ 0.27) (7, 10).

### Statistics

For data analysis, the computer software package SPSS (version 25, IBM Corp., Armonk, NY) was employed. Unpaired categorical and binary variables were analyzed in contingency tables using Fisher’s exact test. The Mann-Whitney U-test was chosen to compare continuous variables as the data were mostly not normally distributed. Results with p<0.05 were considered statistically significant. Group comparisons (BM derived by breast cancer *vs*. BM of other origins, singular BM *vs*. multiple metastatic intracranial lesions, CRP value of < 5 mg/l *vs* >5 mg/l, normal/decreased WBC *vs*. increased WBC, age-adjusted CCI < 10 *vs* CCI > 10, least frail *vs*. moderately-frail *vs*. frailest) regarding OS were conducted using Kaplan-Meier methods with the Gehan-Breslow-Wilcoxon test. A Cox regression proportional hazard model was used to identify potential independent predictors for poor survival in geriatric patients undergoing surgical treatment for BM.

## Results

### Patient Characteristics

Between 2013 and 2018, a total of 180 elderly patients underwent surgery for BM at the authors’ institution. Thereof, 69 patients (38%) were female, 111 patients (62%) were male. Median age was 73 years.

BM most commonly originated from cancers of the lung (n=67, 37%), the gastrointestinal tract (n=31, 17%), melanoma (n=24, 13%), and the breast (n= 17, 9%). At the time of surgical treatment, 57 of 180 patients (32%) already exhibited multiple intracranial BM. The surgically treated BM was located supratentorially in 119 patients (66%), while 61 patients (34%) suffered from infratentorial location of BM. A coagulation-compromising medication was present in 58 patients (32%) at the time of indication. Median OS (mOS) was 7 months (95% CI 4.7-9.3) in geriatric patients suffering from surgically treated BM. Further details are given in **Table 1**.

**Table 1:** Patient characteristics.

	Geriatric patients with surgically treated BM (n = 180)
median age (in yrs)	73
female sex	69 (38%)
primary tumor location	
Lung	67 (37%)
Gastrointestinal	31 (17%)
Melanoma	24 (13%)
Breast	17 (9%)
Other	41 (%)
multiple intracranial metastases	57 (32%)
supratentorial location	119 (66%)
KPS ≥ 70%	153 (85%)
age-adjusted CCI ≤ 10	42 (23%)
frailest (mFI ≥ 0.27)	39 (22%)
anticoagulation medication prior surgery	58 (32%)
in-hospital mortality	7 (4%)
median OS (in months)	7 (95% CI 4.7-9.3)

CCI, Charlson Comorbidity Index; KPS, Karnovsky Performance Score, mFI, modified Frailty Index; OS, overall survival.

#### Influence of Primary Site of Cancer, Tumor Burden and BM Location

**Figure 1:**
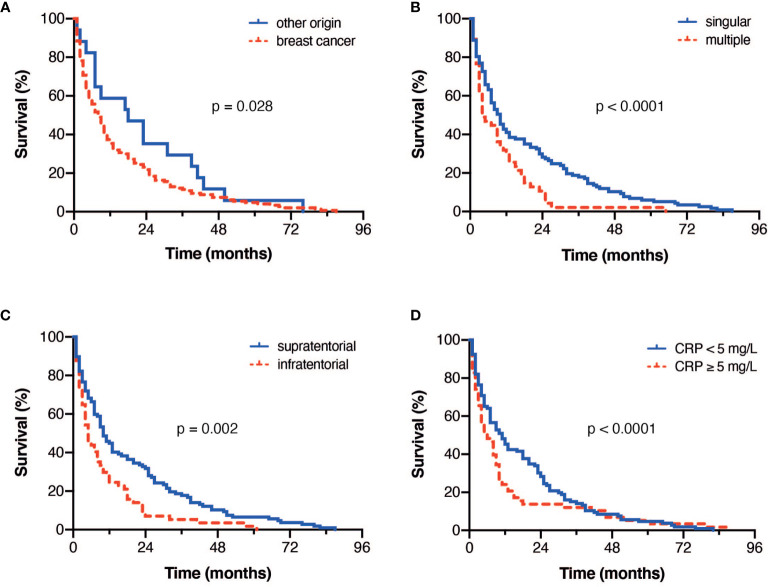
Kaplan Meier-survival curves stratified by **(A)** cancer of breast *versus* other origin **(B)** singular *versus* multiple metastatic lesions **(C)** supra- *versus* infratentorial BM localization **(D)** and CRP value cut-off as indicated.

Patients suffering from BM derived from breast cancer achieved significant longer survival postoperatively with a mOS of 18 months (95% CI 3.8-32.1) compared to patients with BM of other origins (7 months, 95% CI 4.7-9.2; p=0.028; **Figure 1A**).

Furthermore 123 (68%) patients suffered from singular BM, whereas 57 (32%) exhibited multiple intracranial metastatic manifestation prior surgery. Elderly patients with singular BM achieved a mOS of 9 months (95% CI 6.9-11.1), whereas patients suffering from multiple metastatic intracranial lesions achieved a mOS of 4 months (95% CI 2.8-5.1; p<0.0001; **Figure 1B**). Regarding the location of the surgically treated BM, elderly patients with a supratentorial location of BM achieved a mOS of 9 months (95% CI 6.8-11.2), while patients diagnosed with an infratentorial BM location achieved a mOS of 4 months (95% CI 2.5-5.5; p=0.002; **Figure 1C**).

#### Influence of Preoperative Inflammatory Values

In case of a CRP value of <5 mg/l (n=110, 61%) at the time of preoperative admission, elderly patients with BM achieved a mOS of 10 months (95% CI 6.917-13.083). Patients with elevated CRP levels (> 5 mg/l, n=70, 39%) demonstrated a mOS of 4 months during follow-up (95% CI 2.1-5.9). This difference was statistically significant (p<0.0001, **Figure 1D**).

With regard to preoperative WBC count, there was no significant difference in survival when comparing a normal/decreased WBC (mOS 7 months, 95% CI 4.4-9.9) to an increased WBC count (mOS 6 months, 95% CI 3.0-8.9; p=0.6).

#### Influence of Patients Comorbidities on Overall Survival

In the case of a demeaned comorbidity burden (measured by age-adjusted CCI < 10), elderly patients with BM achieved a mOS of 10 months (95% CI 7.629-12.371). Patients with an increased burden of comorbid disease achieved a mOS of 6 months (95% CI 3.6-8.3). This difference did not reach statistical significance (p=0.5).

#### Influence of Frailty on Overall Survival

**Figure 2:**
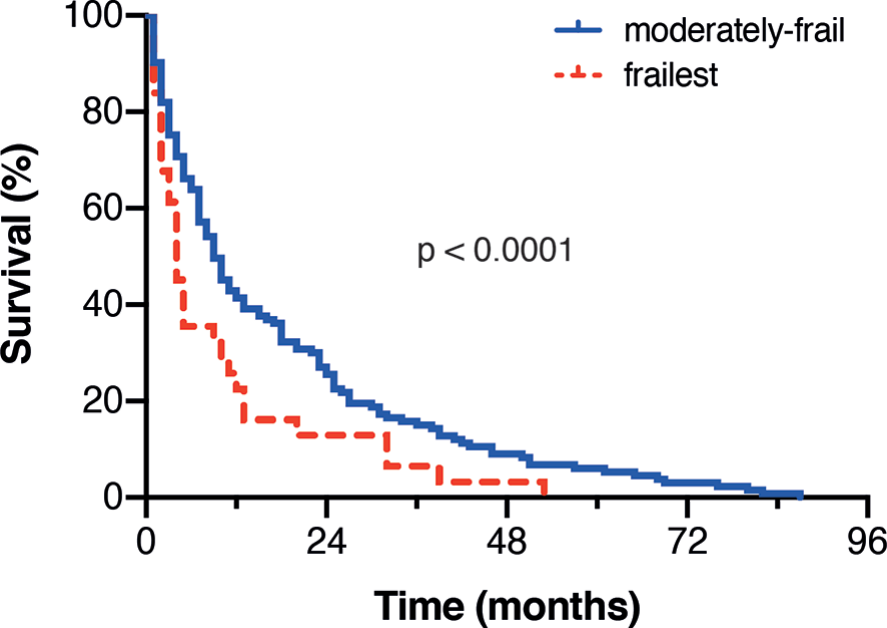
Kaplan Meier-survival curves stratified by the extent of frailty.

Patients preoperatively classified as “least-frail” by means of mFI achieved a mOS of 18 months (95% CI 7.1-28.8) following surgery. Patients categorized as “moderately-frail” based on preoperative mFI achieved a mOS of 7 months (95% CI 5.0-9.0). In contrast, patients who were preoperatively classified as “frailest” reached a mOS of 3 months (95% CI 1.5-4.5; p<0.0001; **Figure 2**).

### Multivariate Analysis

**Figure 3:**
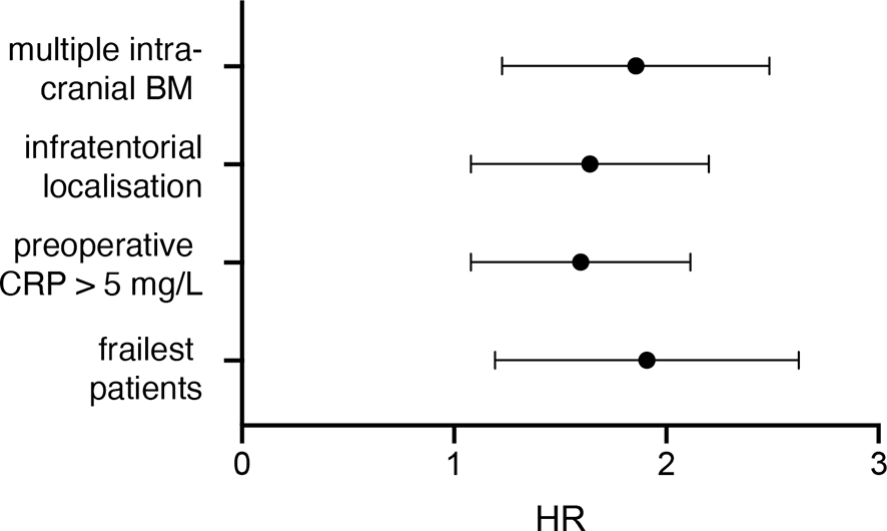
Visualization of the multivariate analysis.

Multivariate analysis was performed using a Cox regression proportional hazard model to identify independent predictors for poor survival in geriatric patients undergoing surgical treatment for BM. The multivariate analysis identified “multiple intracranial BM” (p=0.001, HR 1.78, 95% CI 1.268-2.521), “infratentorial localization” (p=0.011, HR 1.58, 95% CI 1.112-2.227), “preoperative CRP >5 mg/l” (p=0.01, HR 1.54, 95% CI 1.110-2.140) and “frailest patients (mFI ≥ 0.27)” (p=0.002, HR 1.82, 95% CI 1.242-2.665) as independent predictors for reduced OS in elderly patients with surgically treated BM (**Figure 3**).

## Discussion

The increasing emphasis on differentiation of geriatric neurosurgery is particularly important in patients with underlying malignancy. Distinguishing between general and geriatric-focused neurosurgery in the risk assessment and thus in the management of these patients is an important step forward in providing optimal patient-centered care (6). There is an increasing scientific focus on older patients and their specific risk factors to improve preoperative assessment, adjustment of surgical goals, and postoperative care for the benefit of the patient. In an elderly patient population with brain metastasis requiring surgery, this study identifies the presence of multiple BM, infratentorial location of BM, preoperatively elevated CRP levels, and frail condition of the patient as independent predictors of worse overall survival.

As in the present series, diagnosis of multiple BM represents a predictive value for poor survival in various studies (11–13). Surgical resection only removes a part of the intracranial tumor burden, and the target area for postoperative radio/chemotherapy is larger, especially for radiosurgery, which in turn might be associated with an increased postinterventional risk (14).

In addition to the possibility of hydrocephalus or compromise of core structures, the localization of a BM in the posterior fossa is often associated with dysphagia (15, 16). The impaired swallowing function with the risk of aspiration contributes to a possible worsening both immediately postoperatively as well as during further treatment (15).

Elevated CRP levels associated with a chronic inflammatory response have been associated with poor survival in various malignancies (17). Most recently, the influence of preoperative inflammatory parameters (including CRP) on overall survival had been implicated in melanoma patients (18). Whether CRP exerts a direct immunosuppressive role by binding T cells is still subject of further research (19). Hubbard et al. have also shown that in elderly patients, laboratory inflammatory values, such as CRP, increase with advancing frailty (20). One possible explanation for this noted association is that excessive and uncontrolled oxidative stress (which accumulates with age) may represent a core mechanism leading to age-associated frailty (20).

Frailty is a state of vulnerability that reduces the ability to restore physical equilibrium after a stressful event, such as surgery (21). It unfolds as a result of cumulative deterioration and increases the risk of adverse outcomes. Addressing the phenomenon of frailty and its consequences is essential for an optimal decision-making process in the treatment of elderly patients - beyond the scope of single disciplines (6). Numerous instruments have been established and validated to assess frailty as objectively as possible (22). In the present study, such an instrument based on eleven items was used (23). Interestingly, a more subjective assessment of frailty (e.g. using the Clinical Frailty Scale) as a way of enhancing the individual assessment of the treating physician, independently of any coding mechanisms (24). However, such an assessment, which may only be documented to a limited extent, is unfortunately seldomly suitable for retrospective analyses (24). In the present study, patients who were rated as “frailest” on the basis of the mFI had an average mOS of just 3 months. Of note, CCI was not found to be a statistically significant predictor in our analysis, emphasizing that the term frailty conveys more than just an increased comorbidity burden/transcends the predictive power of just an increased comorbidity burden. However, this might derive from the retrospective design and the inherent preselection of operable patients. Nevertheless, this finding highlights the tremendous importance of frailty in preoperative risk assessment before neurosurgical treatment of BM. The intention should not be to deny a possible treatment option, but rather to specifically in an informed manner address the impending risk of a potential postoperative deterioration and/or the occurrence of adverse events that might preclude further necessary cancer treatment components. This information might support the preoperative decision-making process on the course of treatment by the affected patient as well as by family and relatives.

### Limitations

Besides the inherent constraint of the retrospective design of this study, an additional relevant shortcoming is the selection of the measurement instrument of frailty. The multitude of established ways to measure frailty complicates the comparability of different studies. In the present study, a rather objective method of measurement was chosen, which bears the disadvantage of the lack of a physician’s judgment. Malignancy- and treatment-specific factors might bias objective assessment of frailty. Ideally, frailty-specific items as outlined in the **Supplementary Table S1** should have been determined at the earliest possible stage at about the first diagnosis of the underlying malignancy. In the present study items were assessed prior to the resection of the BM which might have negative impact on the severity of frailty. Furthermore, the selected patients were not randomized, but treated according to the recommendations of an interdisciplinary consortium on an individual basis. Nevertheless, the present study identifies factors influencing poor survival in the important and vulnerable patient population of elderly patients with surgically treated BM that have been too sparsely addressed in the literature so far.

## Conclusions

The present study identifies multiple BM, infratentorial location of the metastatic lesion, elevated initial CRP-levels and advanced frailty scoring as risk factors for worsened OS in elderly cancer patients that undergo surgery for BM. These findings might enable to preoperatively identify high risk patients that require special attention in clinical and surgical management and might improve patient counselling and informative patient guidance.

## Data Availability Statement

The original contributions presented in the study are included in the article/**Supplementary Material**. Further inquiries can be directed to the corresponding author.

## Ethics Statement

The studies involving human participants were reviewed and approved by Ethics Committee of the University Hospital of Bonn. Written informed consent for participation was not required for this study in accordance with the national legislation and the institutional requirements.

## Author Contributions

Conceptualization, MH, PS and MS. Data curation, MH, PS and MS. Formal analysis, MH, CS, LE, PS and MS. Writing—original draft preparation, MH, PS and MS. Writing—review and editing, MH, NS, CB, VE, LE, FG, EG, AJ, Y-DK, JL, FL, AR, CS, KS, JW, HV, UH, PS, and MS. Visualization, PS and MS. Supervision, PS and MS. All authors contributed to the article and approved the submitted version.

## Conflict of Interest

The authors declare that the research was conducted in the absence of any commercial or financial relationships that could be construed as a potential conflict of interest.

## Publisher’s Note

All claims expressed in this article are solely those of the authors and do not necessarily represent those of their affiliated organizations, or those of the publisher, the editors and the reviewers. Any product that may be evaluated in this article, or claim that may be made by its manufacturer, is not guaranteed or endorsed by the publisher.
