# Supplementary material for: Outcome of Elderly Patients With Surgically Treated Brain Metastases
Source: Front Oncol. 2021 Jul 26;11:713965. doi: 10.3389/fonc.2021.713965 (PMC8350563; doi:10.3389/fonc.2021.713965)
Supplement: Supplementary file 1 [file Table_1.docx]

***Supplementary Table S1: Overview of the eleven items integrated into the modified frailty index^⁕^***

| **Index weight** | **Description** |
| --- | --- |
| 1 | functional health status prior surgery (only dependent) |
| 1 | history of diabetes mellitus |
| 1 | history of severe COPD/current pneumonia |
| 1 | congestive heart failure |
| 1 | history of myocardial infarction |
| 1 | previous percutaneous coronary intervention; previous cardiac surgery; history of angina |
| 1 | hypertension requiring medication |
| 1 | impaired sensorium |
| 1 | history of transient ischemic attack |
| 1 | cerebrovascular accident/stroke with neurologic deficit |
| 1 | History of revascularization for peripheral vascular disease |

COPD, chronic obstructive pulmonary disease

⁕ modified from Schneider et al. [7]
